# Development and field testing of a decision aid to facilitate shared decision making for adults newly diagnosed with attention‐deficit hyperactivity disorder

**DOI:** 10.1111/hex.13393

**Published:** 2021-12-02

**Authors:** Yumi Aoki, Takashi Tsuboi, Yoshikazu Takaesu, Koichiro Watanabe, Kazuhiro Nakayama, Yasuhito Kinoshita, Mami Kayama

**Affiliations:** ^1^ Graduate School of Nursing St. Luke's International University Chuo‐ku Tokyo Japan; ^2^ Department of Neuropsychiatry, School of Medicine Kyorin University Mitaka‐shi Tokyo Japan; ^3^ Department of Neuropsychiatry, Graduate School of Medicine University of the Ryukyus Okinawa Japan

**Keywords:** attention‐deficit hyperactivity disorder, decision aid, pre–post test, shared decision making, treatment decision making

## Abstract

**Background:**

The number of individuals who are diagnosed with attention‐deficit hyperactivity disorder (ADHD) during adulthood has increased in recent years. However, there is still no decision aid (DA) to help adults newly diagnosed with ADHD make decisions regarding further treatments.

**Objective:**

This study aimed to describe the development process of a DA for adults newly diagnosed with ADHD and its field testing during the shared decision‐making (SDM) process in a clinical setting.

**Methods:**

The development process involved the creation of a DA prototype using the International Patient Decision Aid Standards criteria and revision of the prototype through the stakeholders' reviews. The field testing of the DA compared scores before and after the SDM process on the service users' knowledge scale, decisional conflict scale and the Conners Adult ADHD Rating Scales.

**Results:**

The developed DA contained options of watchful waiting with own coping skills and pharmacological treatment, which consisted of several kinds of drug options. Fifteen adults newly diagnosed with ADHD participated in the field testing. The participant decision‐making quality outcomes such as their knowledge and decisional conflict improved after the SDM process. ADHD severity did not change.

**Conclusion:**

A DA for adults with ADHD was systematically developed following the international criteria. Field testing indicated that the DA could serve as a tool to facilitate the SDM process. Further research on this DA is necessary before its routine implementation.

**Patient or Public Contribution:**

During the development process of the DA, the service users who had already been diagnosed with ADHD reviewed the DA prototype and provided feedback, which improved the final version of the DA.

## 1 INTRODUCTION

Attention–deficit hyperactivity disorder (ADHD) is a neurodevelopmental disorder characterized by the following behaviour‐related symptoms: inattention, hyperactivity and impulsivity.^1^ Although ADHD had been considered to be limited to childhood, research over the last few decades has provided knowledge that most children with ADHD maintain at least some symptoms into their adulthood.^2^ The prevalence of adult ADHD is estimated at 1.2%–3.2%.^2^, ^3^ Hence, there are a considerable number of adults who are suffering from ADHD symptoms. Moreover, in recent years, the increasing number of workers with depression has become a major social problem. As a background, there appear to be many workers who are secondarily developing depression while trying to cope with struggles related to ADHD symptoms. The societal cost due to the increase in ADHD prevalence in the United States is between $143 billion and $266 billion annually, which is mainly attributed to the low productivity and low income of adults with ADHD who are unemployed or absent from work.^4^ Accordingly, it is urgently necessary to take measures against this issue. Some guidelines in several countries such as in the United Kingdom and Australia recommend lisdexamfetamine or methylphenidate extended‐release oral suspension as the first‐line drug for adults with ADHD.^5^, ^6^ Yet, there is no such guidance for adult ADHD treatment in Japan. Furthermore, due to its behaviour‐related symptoms, it is important to take not only medication treatment but also psychoeducation that promotes behavioural changes. However, even in countries where they follow those existing medication treatment guidelines, when medication should be started and how to make decisions about whether to start taking medication or not has yet to be established.

Medical decision making has left traditional conventional ways, where physicians drive the decision‐making process.^7^ Rather, ‘shared decision‐making’ (SDM) is increasingly promoted as a service user‐centred care in various medical fields, including the mental health field.^8^ Particularly for people with mental health conditions, SDM is a central part of the current recovery movement, which is based on the service users’ right to autonomy and self‐determination.^8^, ^9^ Decision aids (DAs) are clinical tools that aim to facilitate SDM between service users and clinicians for a specific condition with a further treatment decision.^10^ DAs reduce decisional conflict, promote service users' knowledge, improve their satisfaction regarding their choice and ensure the congruency of their choice to their personal values.^11^

A DA for parents of children with ADHD who are faced with the decision about ADHD treatment has been developed and reported.^12^ However, to the best of our knowledge, there are as yet no published tools for adults with ADHD who are faced with the decision of their treatments.

The aim of this study was to describe the development process of a DA for adults newly diagnosed with ADHD and its field testing during the SDM process in a clinical setting.

Our hypotheses in the field testing were that the use of the DA during the SDM process would reduce decisional conflict and increase knowledge regarding ADHD. We also assessed if the DA, which included examples of behavioural changes to cope with ADHD symptoms, would reduce ADHD severity.

The DA developed in this study is anticipated to improve the quality of initial care for adults newly diagnosed with ADHD. This service user involvement approach might have a potential positive impact on the abilities of these adults to cope with ADHD symptoms.

## 2 METHODS

### 2.1 Development, review and revision of a DA prototype

The content of a DA prototype was incorporated by findings from the qualitative need's assessment interviews, which were already reported elsewhere,^13^ and researchers developed the prototype in accordance with the quality criteria established by International Patient Decision Aid Standard.^14^ The prototype was then reviewed by stakeholders, such as service users and health professionals. Individuals diagnosed with ADHD during adulthood were approached at follow‐up appointments of the outpatient service. Health professionals who regularly see adults with ADHD at the same outpatient service were also approached. They were welcome to write any comments directly on the prototype while reviewing it. The readability and understandability of the DA prototype, including the length of explanation, amount of information and balance of related information were assessed. Trends in responses and narrative feedback were collected, and similar comments were assembled. The researchers then discussed the input from the stakeholders, which was used to improve the DA prototype.

### 2.2 Field testing the DA

The developed DA was then field‐tested during the SDM process in a clinical setting described below.

#### 2.2.1 SDM programme for psychiatric outpatients

We conducted field testing of the DA using a clinical SDM process that we developed and reported previously.^15^ The SDM programme comprises the three steps as described below.

Step 1. *Initial consultation*: *Option presentation consultation*: After the psychiatrist informs the adult of the diagnosis as ADHD, further treatment options such as watchful waiting with coping skills or additional medication with coping skills are shared between the adult and the psychiatrist using the DA booklet.
Step 2. *Deliberation outside the service*: The participant goes home with the DA, and freely reviews and considers it.
Step 3. *Decision‐making consultation*: At the next appointment, the participant sees the psychiatrist. Both parties discuss the treatment options using the DA and finally decide which treatment can be further taken by the participant.

#### 2.2.2 Participants

Participants were recruited from three outpatient psychiatric services in Tokyo from March 2019 to February 2020. For the inclusion criteria, we included those who visited the psychiatric services and met the following criteria: (i) outpatients aged 20 years or older; (ii) received a first‐time ADHD diagnosis followed by Diagnostic and Statistical Manual of Mental Disorders Fifth Edition (DSM‐5).^1^ The exclusion criteria were as follows: (i) participants who have already been diagnosed with ADHD; (ii) current substance dependence and abuse; and (iii) unable to speak Japanese. Participants requiring hospitalization in the psychiatric ward due to symptom severity or suicidality were also excluded and referred to applicable psychiatric services. Seventeen individuals who fulfilled the inclusion criteria were approached after the initial consultation. The sample size was selected in accordance with the methods used in the DA literature for field testing.^16^, ^17^, ^18^ Two of the 17 individuals withdrew their agreements immediately after providing the written consent: one moved to another service and the other could not revisit the service owing to a weather disaster. Participants filled out the questionnaires immediately after the initial consultation (baseline), immediately after the decision‐making consultation, and in the 1‐ to 2‐month follow‐up at the outpatient services.

#### 2.2.3 Measurements

Baseline—filled the questionnaires immediately after the initial consultation

*(a) Preferences for roles in decision making*: We used the Control Preference Scale (CPS)^19^ to assess the participants’ preferences for roles in decision making. We used the Japanese translation version of the CPS. The CPS comprises the following items: (i) clinician should ‘make the decision using all that is known about the medicines’; (ii) clinician should ‘make the decision but strongly consider [patient's] opinion’; (iii) patient and clinician should ‘make the decision together, on an equal basis’; (iv) patient should ‘make the decision but strongly consider the clinician's opinion’; and (v) patient should ‘make the decision using all the patient knows or learns about the medicines’. The previous study using the Japanese version of the CPS demonstrated inter‐rater reliability with a substantial level of agreement (*κ* coefficients = 0.61).^20^

(b) *Decisional conflict*: We assessed decisional conflict, defined as uncertainty about the course of action to take when choosing among several medical procedures,^21^ using the Japanese version of the decisional conflict scale (DCS).^22^ The DCS comprises 16 items and the following five subscales: feeling informed, having clear values about benefits and risks, support to take the decision, uncertainty, and perceived effectiveness of the decision. The items are scored from 0 (strongly agree) to 4 (strongly disagree), with higher scores indicating a higher decisional conflict. The total scores range from 0 (no conflict) to 100 (maximal conflict). Scores lower than 25 are associated with implementing decisions, and those above 37.5 are associated with decision delay or feeling unsure about implementation.^21^ The scores on the subscales of the Japanese version of DCS demonstrated high internal consistency (Cronbach's *α* = .84–.96).^22^

(c) *Knowledge*: Participants' knowledge regarding ADHD and its treatment was measured using a questionnaire containing 18 items with the response options ‘true’, ‘false’, and ‘don't know’. Of the 18 items, 13 items were presented in the DA and 5 items were not. The number of items, which were answered correctly within each subgroup, that is, the items presented in the DA and those not presented in the DA, was calculated. We developed this questionnaire for this study based on knowledge measures used in the previous ADHD study^12^ with the developer's permission. We used 13 items from measures in Brinkman et al.^12^ and 5 of our own items. Medication treatment options available for ADHD vary widely from country to country. Our own items were based on information regarding medication treatment options currently approved and marketed in Japan. The questionnaire was reviewed by the authors, including ADHD experts for content validity.

(d) *ADHD severity*: We assessed ADHD severity using Conners' Adult ADHD Rating Scales‐Self‐Report: Long version (CAARS‐S: L).^23^, ^24^, ^25^ The CAARS‐S: L includes 66 questions and consists of eight subscales: inattention/memory problems, hyperactivity/restlessness, impulsivity/emotional lability, problems with self‐concept, DSM‐IV inattentiveness symptoms, DSM‐IV hyperactivity–impulsivity symptoms, DSM‐IV ADHD symptoms total and ADHD Index. The answers were evaluated using a 4‐point Likert scale with four options: 0: almost never, never; 1: occasionally, sometimes; 2: most of the times, usually; 3: very often, always. We used the Japanese version of CAARS‐S: L.^23^, ^24^, ^25^ The scores on the subscales of the Japanese version of CAARS‐S: L demonstrated good internal consistency (Cronbach's *α* = .73–.88).^25^

Post—filled the questionnaires immediately after decision‐making consultation

(a) *How decisions were made*: We used the scales developed by Strull et al.^19^ to assess how the decision was made. The scales comprise the following items: (i) clinician ‘made the decision using all that is known about the medicines’; (ii) clinician ‘made the decision but strongly consider [patient's] opinion’; (iii) clinician and patient ‘made the decision together, on an equal basis’; (iv) Patient ‘made the decision but strongly consider the clinician's opinion’; and (v) patient ‘made the decision using all [the patient] knows or learns about the medicines’.

(b) *Decisional conflict*: We assessed decisional conflict using the Japanese version of the DCS.^22^

(c) *Knowledge*: Participants' knowledge regarding ADHD and its treatment was measured using exactly the same questionnaire that was administered after the initial consultation.

One‐ to 2‐month follow‐up

2.2.4

*ADHD severity*: Within the 1–2‐month follow‐up after the decision‐making consultation, we assessed ADHD severity using the Japanese version of CAARS‐S: L^24^, ^25^ as a long‐term follow‐up.

#### 2.2.5 Data analysis

We assessed the concordance between preferences for a role in decision‐making and how decisions were made. We combined the three categories reflecting participant involvement into one category and contrasted it with the categories of the clinician‐alone and patient‐alone decision‐making. Gwet's Agreement Coefficient (AC_1_) was used to determine the concordance.

For the knowledge, decisional conflict and ADHD severity, we used a paired *t*‐test to evaluate for significant differences between the before‐ and after‐SDM process if the data were normally distributed. Otherwise, we used a Wilcoxon signed‐rank test. The mean score and associated standard deviation (SD) were also calculated.

This study protocol was approved by the Ethics Board at St. Luke's International University (18‐A055).

## 3 RESULTS

### 3.1 Development, review and revision of a DA prototype

The developed DA prototype consisted of a 20‐page A5 paper prepared as a booklet. The needs assessment interviews suggested that individuals diagnosed with ADHD during adulthood experienced difficulties in accepting their diagnosis and had identity concerns just after the diagnosis.^13^ Therefore, in the DA prototype we attempted to provide appropriate evidence‐based information about ADHD to promote self‐understanding and reduce stigmatized attitudes towards ADHD. The DA prototype then contained options of watchful waiting with own coping skills and pharmacological treatment, which consisted of several kinds of drug options. Table 1 shows the contents and rationales of the DA prototype.

**Table 1:** Contents and rationales of the decision aid for adults newly diagnosed with ADHD

Content	Pages	Rationale/reference
*1. What is ADHD?*		IPDASi Qualifying Criteria: The patient decision aid describes the health condition or problem for which the index decision is required
Examples of difficulties and burdens in the workplace experienced by adults with ADHD	6, 7	Findings from interviews as needs assessment (Aoki et al. BMC Psychiatry, 20: 373, 2020)
Objective information such as diagnostic criteria, prevalence rate, comorbidity	8, 9	‐ DSM‐5, 2013 ‐ Higuchi et al. Adult ADHD treatment guide, JIHO, 2013 (in Japanese) ‐ Kessler et al. Am J Psychiatry, 163(4): 716–23, 2006 ‐ Simon et al. Br J Psychiatry, 194(3): 204–11, 2009 ‐ Saito. RINSHO SEISHIN IGAKU, 46(10): 1233–42, 2017 (in Japanese)
Explanation of fluctuation and changeability using coping skills	10	Findings from interviews as needs assessment (Aoki et al. BMC Psychiatry, 20: 373, 2020)
*2. Acquiring coping skills*		
Examples of coping skills	12–14	Findings from interviews as needs assessment (Aoki et al. BMC Psychiatry, 20: 373, 2020)
*3. Deliberating and deciding on further treatment*		IPDASi Qualifying Criteria: The patient decision aid explicitly states the decision that needs to be considered (index decision)
*3.1. Options: watchful waiting with coping skills and watchful waiting with coping skills plus medication treatment*		IPDASi Qualifying Criteria: The patient decision aid describes the options available for the index decision
A table comparing each option (advantages, disadvantages, consequences)	16	IPDASi Qualifying Criteria: The patient decision aid describes the positive features of each option IPDASi Qualifying Criteria: The patient decision aid describes the negative features of each option IPDASi Qualifying Criteria: The patient decision aid describes what it is like to experience the consequences of the options ‐Okada. SEISHIN IGAKU, 59(3): 253–8, 2017 ‐Watanabe. Nervous system agents in KON‐NICHI NO CHIRYOYAKU, NANKO‐DO, 2019
A values clarification exercise with a 5‐point Likert scale	17	IPDASi Qualifying Criteria: The patient decision aid asks patients to think about which positive and negative features of the options matter the most to them
*3.2. Options: methylphenidate extended‐release oral suspension and atomoxetine hydrochloride*		IPDASi Qualifying Criteria: The patient decision aid describes the options available for the index decision
A table comparing each option (advantages, disadvantages, consequences)	18	IPDASi Qualifying Criteria: The patient decision aid describes the positive features of each option IPDASi Qualifying Criteria: The patient decision aid describes the negative features of each option IPDASi Qualifying Criteria: The patient decision aid describes what it is like to experience the consequences of the options ‐ Okada. SEISHIN IGAKU, 59(3):253‐8, 2017 ‐ Watanabe. Nervous system agents in KON‐NICHI NO CHIRYOYAKU, NANKO‐DO, 2019 ‐ The prescribing information of ‎methylphenidate hydrochloride ‐ The prescribing information of atomoxetine
A values clarification exercise with a 5‐point Likert scale	19	IPDASi Qualifying Criteria: The patient decision aid asks patients to think about which positive and negative features of the options matter most to them
Memo field to prepare for decision‐making consultation	20	IPDASi Qualifying Criteria: The patient decision aid includes tools like worksheets or lists of questions to use when discussing options with a practitioner

Abbreviations: ADHD, attention‐deficit hyperactivity disorder; DSM‐5, Diagnostic and Statistical Manual of Mental Disorders Fifth Edition; IPDASi, International Patient Decision Aid Standards instrument.

Five service users then reviewed the DA prototype (Supporting Information Appendix S1). There were some comments unique to ADHD (e.g., too colourful/decorative to concentrate on reading). We then avoided unnecessary embellishment and used only two colours. For the description of condition and coping skills, the service users gave additional examples from their own experiences. We referred to these additional examples. Five psychiatrists also reviewed the DA prototype (Supporting Information Appendix S2). Similarly, to the service users, there were suggestions to hesitate using decorations and many colour fonts. Although psychiatrists were familiar with percentage and pie chart, we did not address them because we adopted a more user‐friendly description. A single cycle of review and revision occurred for both the service users and the psychiatrists. Thereafter, through the process of the stakeholders' reviews, the final version of the DA was developed. Supporting Information Appendix S3 shows the final DA containing both the Japanese version and the English version.

### 3.2 Field testing the DA

#### 3.2.1 Summary of participants

Participants were 15 adults newly diagnosed with ADHD. The mean age was 42.4 years. Table 2 shows the demographic characteristics and treatment decision‐making of the participants. There were no participants who dropped out during the SDM process. Accordingly, all the participants experienced the three steps in SDM: the option presentation consultation, deliberation outside the service and the decision‐making consultation.

**Table 2:** Demographic characteristics of the study participants—field testing

Characteristics	*n* = 15
Sex, female, *n* (%)	6 (37.5)
Mean age (years) (SD)	42.4 (12.0)
Comorbid mental health conditions, yes, *n* (%)	7 (46.7)
Comorbid mental health conditions, *n* (%)^a^	
Depression	4 (26.7)
Bipolar disorder	1 (6.7)
Anxiety disorder	1 (6.7)
Panic disorder	1 (6.7)
Eating disorder	1 (6.7)
Sleep disorder	2 (13.3)
Autism spectrum disorder	4 (20.0)
Occupation, *n* (%)	
Full‐time job	7 (46.7)
Part‐time job	3 (20.0)
Freelance	2 (13.3)
Employed under disability status	1 (6.7)
Housewife	2 (13.3)
Treatment decision‐making for ADHD, *n* (%)	
Watchful waiting with own coping skills	9 (60.0)
Own coping skills plus ADHD medication	6 (40.0)

Abbreviation: ADHD, attention‐deficit hyperactivity disorder.

^a^ Includes multiple responses.

#### 3.2.2 Concordance between role preferences and experienced decision

Regarding preferences, 14 (93%) of participants preferred patient involvement in decision making, while only 1 (7%) preferred clinician‐alone decision‐making and none preferred patient‐alone decision‐making. Regarding practical experiences, 14 (93%) reported that they were involved in the decision‐making, while only 1 (7%) answered that the clinician made the decision, and none chose patient‐alone decision‐making. The concordance between role preferences and experienced decisions (15 pairs) was AC_1_ = 0.88 (0.60–1.09). This indicated that it was more than a coincidence.

#### 3.2.3 Participant decision‐making quality outcomes

(a) *Decisional conflict*: The participants' mean total decisional conflict score at baseline was 56.4 (±20.0). After the SDM process, the mean score was 32.8 (±14.4) (*p* = 0.001), which indicates that their conflicts significantly improved, and they were feeling more confident about their decisions. All subscales were >37.5 at baseline, indicating that the participants were feeling unsure about the implementation of each category (informed, value clarity, support, uncertainty and effectiveness); however, they all significantly improved after the SDM process. The mean subscales, other than ‘uncertainty’, after the SDM process were <37.5 (Table 3).

**Table 3:** Participant decision‐making quality outcomes

	Baseline	Post‐SDM	Mean change	*p*‐value
Knowledge (correct items), mean (SD)				
Specific items in DA (13 items)	5.8 (3.3)	9.7 (2.2)	3.9	0.001^a^
Not in DA (5 items)	1.1 (1.0)	2.4 (1.0)	1.3	0.003^b^
Overall (18 items)	6.9 (4.1)	12.1 (2.6)	5.2	<0.001^a^
DCS (range 0–100), mean (SD)				
Informed subscale	54.4 (24.2)	25.5 (14.2)	−28.9	0.003^a^
Value clarity subscale	56.1 (28.6)	35.6 (22.0)	−20.5	0.047^a^
Support subscale	49.4 (25.1)	23.3 (16.1)	−26.1	0.001^a^
Uncertainty subscale	66.7 (21.1)	42.8 (24.2)	−23.9	<0.001^a^
Effectiveness subscale	55.4 (23.4)	35.8 (19.7)	−19.6	0.002^b^
Overall	56.4 (20.0)	32.8 (14.4)	−23.6	0.001^b^

*Note*: The results shown are based on 15 participants who had complete data at baseline and post‐SDM.

Abbreviations: DA, decision aid; DCS, decisional conflict scale; SD, standard deviation.

^a^ Paired *t*‐test.

^b^ Wilcoxon signed‐rank test.

(b) *Knowledge*: The participants' mean knowledge score of the specific items mentioned in the DA improved from 5.8 (±3.3) at baseline to 9.7 (±2.2) after the SDM process (*p* = 0.001). The participants’ mean knowledge score of the items not mentioned in the DA also improved after the SDM process (*p* = 0.003) (Table 3).

#### 3.2.4 ADHD severity

There was no significant change between the baseline and the 1–2‐month follow‐up regarding any subscale (Table 4).

**Table 4:** ADHD severity

	Baseline	1–2 months	Mean change	*p*‐value
CAARS self‐rating, mean (SD)				
Inattention/memory problems	21.9 (9.4)	21.4 (8.6)	−0.5	0.738^a^
Hyperactivity/restlessness	15.6 (5.7)	16.3 (6.6)	0.7	1.000^b^
Impulsivity/emotional lability	17.2 (8.0)	16.3 (7.3)	−0.9	0.715^a^
Problems with self‐concept	13.2 (4.3)	13.5 (4.3)	0.3	0.737^a^
DSM‐IV: Inattentiveness symptoms	14.8 (7.0)	14.3 (7.2)	−0.5	0.581^a^
DSM‐IV: Hyperactivity–impulsivity symptoms	9.9 (4.3)	9.2 (3.8)	−0.7	0.553^a^
DSM‐IV: ADHD symptoms total	25.7 (12.0)	28.2 (11.2)	2.5	0.498^a^
ADHD Index	19.0 (7.5)	19.7 (7.5)	0.7	0.654^a^

*Note*: The results shown are based on 13 participants who had complete data at baseline and post‐SDM.

Abbreviations: ADHD, attention‐deficit hyperactivity disorder; SD, standard deviation.

^a^ Paired *t*‐test.

^b^ Wilcoxon signed‐rank test.

## 4 DISCUSSION

To our knowledge, this is the first study to develop a DA for adults newly diagnosed with ADHD and to conduct field testing of the DA during the SDM process in a clinical setting.

The findings indicate that adults with ADHD are able to participate in the SDM process while acquiring the relevant knowledge and assessing their preferences. The concordance between their role preferences and experienced decisions also indicated that adults with ADHD were successfully involved. Moreover, the significant decrease of decisional conflict, after the SDM process, suggested that the participants made effective decisions regarding further treatments. Ramon et al.,^26^ who developed an SDM programme for medication for schizophrenia, depression or bipolar disorder, reported participation in treatment decision‐making brought a significant decrease in decisional conflict after the SDM process. Paudel et al.^27^ also developed an SDM programme for community mental health services in treating severe mental illnesses and revealed that decisional conflict had significantly improved after the SDM. Thus, the present study contributes to the existing SDM literature in which people with mental health conditions were able to participate in their own decision‐making and make effective decisions.

However, this study found no change in the ADHD severity in the 1–2‐month follow‐up. Several reasons may underlie this absence of change. The sample size of the study was small. Also, the 1–2‐month follow‐up might not be appropriate to adequately assess the ADHD severity because the participants were still struggling to acquire new strategies during that period. It takes a little longer for their coping skills to adapt to their environments. Brinkman et al., who conducted an SDM intervention for the parents of children with ADHD, also reported no significant difference in the behaviour rating scale for assessing treatment response.^12^ Furthermore, there were few previous SDM studies in the psychiatric field that found evidence regarding long‐term outcomes. Thus, the SDM process in the initial phase of treatment might not have an effect on the long‐term clinical outcomes.

There were some limitations in this study. First, the sample size was relatively small because we recruited the participants in the services where the SDM programme had been introduced.

Second, because of the exploratory nature of this study as a field testing, the evaluations were not compared with adults newly diagnosed with ADHD who had decided on further treatment without the DA, and who were not involved in treatment decision‐making. As the international criteria^14^ indicate, further studies that will incorporate a control group are necessary. Third, we used the knowledge scale that had not been tested in a Japanese sample before this study. Moreover, the current study was conducted in psychiatric services familiar with SDM; the implementation and widespread usage of this DA in other facilities may require an appropriate SDM training programme. Nonetheless, the findings show that the participants utilized the DA during the SDM process in a clinical setting. There are two ways adults are usually diagnosed with ADHD: (1) they are already receiving treatment for another psychiatric diagnosis (e.g., depression) and then are diagnosed with ADHD, or (2) they are uncertain about their condition and seek ADHD diagnosis on their own.^28^ The latter has been increasing with the spread of informational media on developmental disorders.^29^ Whichever the case, as the results showed, the developed DA during the SDM process in the present study is a practical tool for adults newly diagnosed with ADHD. Furthermore, the pharmacological treatment options of ADHD are rapidly advancing. Our DA will need to be updated in accordance with the latest situation regarding available treatment options.

## 5 CONCLUSION

We systematically developed a DA for adults newly diagnosed with ADHD following the international criteria. The field testing suggests that the successfully developed DA could serve as a tool to facilitate SDM in clinical settings. Further research on this DA is necessary before its routine implementation.

## CONFLICT OF INTERESTS

The authors declare that there are no conflict of interests.

## AUTHOR CONTRIBUTIONS

All authors conceptualized and designed the study. Yumi Aoki secured the grant to conduct the study. Takashi Tsuboi, Yoshikazu Takaesu and Koichiro Watanabe organized data collection. Yumi Aoki carried out the questionnaire surveys. Yumi Aoki, Takashi Tsuboi, Kazuhiro Nakayama, Yasuhito Kinoshita and Mami Kayumi contributed to the analysis and writing of the manuscript. All authors read and approved the final draft.

## Supporting information

Supplementary information.

Supplementary information.

Supplementary information.

## Data Availability

The data that support the findings of this study are available from the corresponding author upon reasonable request.
