# Supplementary material for: Development and field testing of a decision aid to facilitate shared decision making for adults newly diagnosed with attention‐deficit hyperactivity disorder
Source: Health Expect. 2021 Dec 2;25(1):366–73. doi: 10.1111/hex.13393 (PMC8849269; doi:10.1111/hex.13393)
Supplement: Supplementary file 1 — Supplementary information. [file HEX-25-366-s003.pdf]

Appendix 1. Summary of qualitative findings on the decision aid prototype - service users' reviews

Categories	Comments/Suggestions	Responses
Plain language	“SHOGEKISEI (impulsivity)” is difficult to read	Added Japanese subtitle
	“Remind” is difficult to understand	Used a Japanese word instead of “remind”
	“Placebo” is difficult to understand	Avoided using “placebo”
	“Central nervous system” is difficult to understand	Avoided using “central nervous system”
	“Imbalance of brain chemicals” is difficult to understand	Used simple word “DEKO-BOKO” in Japanese
Layout/format	Decorative letters are an eyesore	Avoided decorative letters
	Too colourful	Used only two colours
Missing information	“Easily dozing off” needs a background explanation	Added explanation
	Use of smartphone alarm should be recommended	Added “using smartphone alarm” as a coping skill
	“Going to vending machine a lot” should be “taking a rest a lot”	Replaced with “taking a rest a lot in different places”
	Insurance information is needed	Added insurance information
Clarity of information provided	“My good/strong points” should be “my favourite things”	Replaced with “my favourite things”
	NOT poor/inferior emphasizes rather poor	Avoided using the words “poor” and “inferior”
	“Forgettable” should be “loses things a lot”	Replaced with “loses things a lot”
	“Display in noticeable places” should be “put important things together in a predetermined place”	Replaced with “put important things together in a predetermined place”
Instructions	・Provide instructions on using the DA<br>・Be more explicit on how to use the DA (use during the SDM process with a psychiatrist)	Added instructions to the first part of the DA
Others	Need web version	Electronic version will be considered in the future
