# Supplementary material for: Development and field testing of a decision aid to facilitate shared decision making for adults newly diagnosed with attention‐deficit hyperactivity disorder
Source: Health Expect. 2021 Dec 2;25(1):366–73. doi: 10.1111/hex.13393 (PMC8849269; doi:10.1111/hex.13393)
Supplement: Supplementary file 2 — Supplementary information. [file HEX-25-366-s001.pdf]

Appendix 2. Summary of qualitative findings on the decision aid prototype - psychiatrists' reviews

| Categories | Comments/Suggestions | Responses |
| --- | --- | --- |
| Layout/format | All Chinese characters do not need Japanese subtitles | Subtitles were provided only when the Chinese characters appeared for the first time |
| | Too colourful, too decorative | Avoided decorations and used only two colours |
| | Checkboxes should be inserted before each symptom | Added checkboxes |
| | Prefer pie chart to pictogram | No change: pictogram is recommended for all levels of health literacy |
| | Prefer % to number out of 100 | No change: number out of 100 is recommended for all levels of health literacy |
| Missing information | Explanation of developmental disorder | Added explanation of developmental disorder |
| | How to stop medication | No change because the DA is about whether to take medication or not |
| | Reconsideration is available once decision is made | Added availability of reconsideration |
| | Monthly cost information | Added monthly cost information |
| Clarity of information provided | Need a message that all patients initially try to acquire coping skills and then consider medication options | Added the message |
| | Samples should be provided when asking users to think about their coping skills | Added samples |
