# Supplementary material for: Development and field testing of a decision aid to facilitate shared decision making for adults newly diagnosed with attention‐deficit hyperactivity disorder
Source: Health Expect. 2021 Dec 2;25(1):366–73. doi: 10.1111/hex.13393 (PMC8849269; doi:10.1111/hex.13393)
Supplement: Supplementary file 3 — Supplementary information. [file HEX-25-366-s002.pdf]

● 大人になってADHDとわかった方へ ●

たいしょほう　ちりょうほう

自分にあった対処法・治療法をみつけるための手引き

この手引きについて

ADHDは、日本語では注意欠如（ちゅういけつじょ）・多動症（たどうしょう）と呼ばれます。「授業中動き回る子ども」のように、子ども特有のものと思われがちですが、大人でもその症状をもち、生活に様々な困り事を抱えている人がいることがわかってきました。**社会人になり職場で困り事が重なってわかる人もいるのです。**

ここでは、まず、そうした「社会人のADHD」の特徴と自分でできる工夫や対処法について述べました。さらに、対処法に加えることのできる薬の治療についてまとめました。**自身の特徴を知り、あなたに合った対処法・治療法を、医療者と一緒に選ぶときの手引き**として活用できます。

この手引きの使い方

　この手引きは、ADHDとわかった方が、医療者と話し合いながら、治療法を選ぶためのものです。
手引きを自宅に持ち帰ってよく読み、十分に検討しながら今後の治療の方針を一緒に考えていきます。

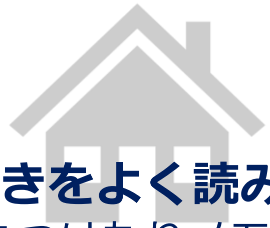

手引きをよく読みます

○をつけたりメモ欄に
記入したりします

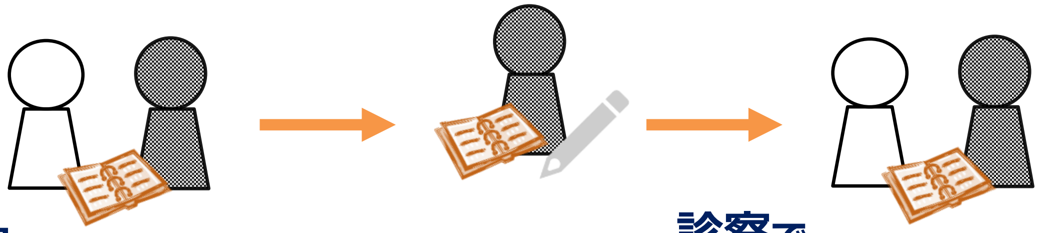

診察で

・現在の状態
・対処法/治療法の選択肢
について確認します

診察で

・あなた：質問する
　医師：回答する
・○をつけた項目や
　メモした内容について
　話し合います
・今後の方針を決めます

※一度で決まらない場合は
また持ち帰って検討します

もくじ

- ADHD について知る …… 5
- 対処法 をみつける …… 11
- 対処法 に加えることのできる 薬の治療 …… 15

ADHDについて知る

こんなことはありませんか？

●上司から指示されて

□ 口頭だけだと頭に残らない（文字があるとよい）

□ 最初に言われたことを思い出せない

□ 一度にたくさん言われると、頭が真っ白

●電話を受けて

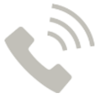

□ 伝言や依頼の内容を聞き誤る

□ 誰からの電話か聞き忘れる

●事務作業で

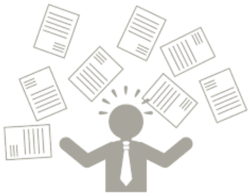

□ 名前や数を間違って入力してしまう

□ 書いてあることを読み飛ばす

□ 指示の意図を理解しないままに取り掛かり、失敗する

□ 上司に『進捗の報告がない』と注意される

●会議で

□ 貧乏ゆすり、ペンをカチカチなど、どこか体が動いている

□ 関心のない話題だと居眠りをしてしまう

こんなことはありませんか？

●同僚との会話で

□『あれどうだった？』と聞かれても思い出せない

□ バレるのに、とっさにウソや適当なことを言ってしまう

□ 興奮しやすい

□ 愛想よくできるけど、あとで疲れる

●デスクワークで

□ すぐ違う場所に行って一息入れたくなる

□ 興味のあること以外は座っているのが苦痛

●段取りの決まっている仕事で

□『勝手な行動をしないで』と注意される

●昇進して

□ 部下の管理がうまくできない

ここにあげたものは、社会人になってADHDとわかった人々の職場での体験です。同じような経験はありますか？

☞ つぎからは、ADHDの特徴についてみていきます。

大人のADHDの特徴

不注意と多動性（たどうせい）/衝動性（しょうどうせい）の2つの症状があります[1]

● **不注意** には

・ケアレスミスをしやすい

・注意を持続することが難しい

・課題を整理することが苦手、課題を最後まで果たせない

・必要なものや日々の活動を忘れがち

・外からの刺激で注意散漫になりがち

などがあります

● **多動性/衝動性** には

・手足をもじもじする、そわそわした動きをする

・座っていることが期待されている場面で席を立つ

・じっとしていられない

・しゃべりすぎる、質問が終わる前にうっかり答え始める

・順番待ちが苦手

・他の人の邪魔をしたり、割り込んだりしがち

などがあります

大人のADHDの特徴

●原因は？

　脳の機能のアンバランス（凸凹デコボコ）や脳の神経伝達物質の働きが関与しているとされています[2)]。

●自分だけ？

　いいえ、決してめずらしいことではありません。

大人100人中 **2～4**人はADHD症状をもつとされています[3)4)]。

●ADHDだけ？[5)]

　大人のADHDの約**7**割が、ほかの精神科疾患も持ち合わせているとされています。とくに、仕事上の困難から、こころのバランスを崩し、うつ病と診断される人が増えています。

おなじ発達障害に分類される自閉スペクトラム症や躁（そう）とうつを繰り返す双極性障害（そうきょくせいしょうがい）をあわせもつ人も多いとされています。

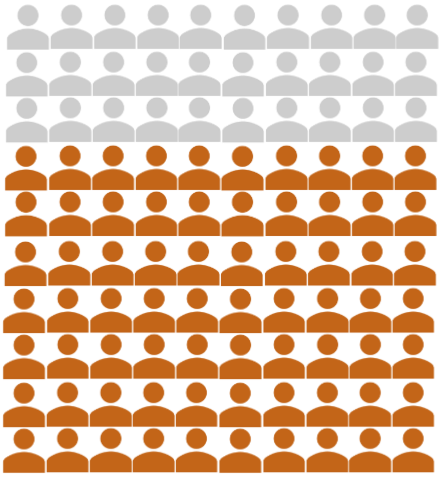

100人中 約**30**人
ほかの精神科疾患 **なし**

100人中 約**70**人
ほかの精神科疾患 **あり**

大人のADHDの特徴

●障害なの？

ADHDは発達障害の１つに分類されますが、障害といっても固定されたものではありません。ADHDの特性によって「仕事などに困難が生じている」ときに問題になるのであり、自身の特性を知り、対処法を工夫することで、症状が目立たなくなることもあります。

ADHDについて、疑問や質問を書いておきましょう

☞ つぎは、対処法をみていきます

対処法をみつける

対処法をみつける

ADHDの特徴を理解したら、つぎは対処法です

1. 自分の特性を知る

まずは自分を知ることです。苦手なことは何ですか？
注意されるのはどんなときでしょう。反対に、好きなことや
得意なこと、楽しいと感じることはどんなことですか？
周りの人に聞いてもいいですね。書いてみましょう。

【苦手なこと】

【好きなこと・やっていて楽しいこと】

2. 対策をたてる

つぎに、対策をたてます。たとえば・・・

- 忘れ物が多い ⇒ 必要な物は1か所にまとめておく
- 失言が多い ⇒ 10秒数えてから発言する
- 期日が守れない ⇒ スマホのリマインダーを使う
- 片付けられない ⇒ しまう場所を決める
使わない物は捨てる
- よいことを思いついた ⇒ 一晩置いてもう一度考える
- 予定を詰め込む ⇒ 合間に空き時間を30分つくる
- 書いたメモをなくす ⇒ ノートを1つ決めて持ち歩く
- 優先度がわからない ⇒ 情報を色分けする、番号を振る

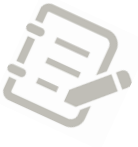

人の力を借りるのも大切です・・・

- ケアレスミスが多い ⇒ ダブルチェックを頼む
- 期日が守れない ⇒ 声かけをお願いしておく
- 指示が覚えられない ⇒ 書きとめ、合っているか聞く

他にはどんなことができそうですか？
自分の特徴を振り返り、対策をたて実践してみましょう

【対策を立てる】

自分にできそうな対処法をあげてみましょう

例. その日にやることを見えるところ貼っておく

対処法に加えることのできる 薬の治療

対処法に加えて 薬を飲む or 飲まない

ADHDは、自分で工夫する、周りの人の力を借りるなどの対処に取り組んだうえで、**薬を飲むという選択肢**もあります。ここでは、薬を飲む場合と飲まない場合とを比べてみます。

	飲む	飲まない
利点	●症状が軽快する[6-9)]<br>穏やかになる・注意が行き届く<br>☞ 生活や仕事がしやすい	●副作用を避けられる<br>●費用の負担がない
欠点	●100人中76~80人が何らかの副作用を経験する※[6)7)]<br>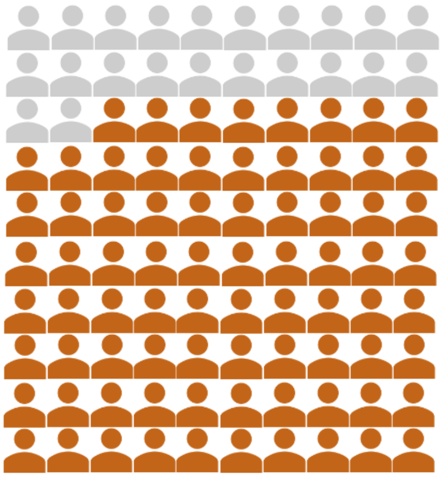<br>副作用なし<br>副作用あり<br>※吐き気,食欲低下, 睡眠障害 など<br>副作用の詳細は**18**頁<br>●費用がかかる<br>（保険種別により支払う金額は異なります）	●対処法のみでは改善されない場合、いまある症状が続く<br>☞ 生活や仕事に支障をきたす

対処法に加えて 薬を飲む or 飲まない

●考えを整理しましょう

あなたにとって、薬に関する以下の内容は、どの位重要ですか?
0~5で重みづけをしてみましょう。

内容	重要でない					重要である
ADHD症状が改善する	0	1	2	3	4	5
仕事や生活がしやすくなる	0	1	2	3	4	5
副作用を避けられる	0	1	2	3	4	5
費用の負担がない	0	1	2	3	4	5

その他（気になること）

•	0	1	2	3	4	5
•	0	1	2	3	4	5
•	0	1	2	3	4	5
•	0	1	2	3	4	5

☞ つぎに、薬を希望する場合の薬の選択肢をみていきます

–薬を希望する方へ-

どちらの薬を選ぶ?

薬には２つの選択肢があります。特徴を比べてみます[6~9])。

	メチルフェニデート塩酸塩徐放錠<br>**コンサータ ®**	アトモキセチン塩酸塩<br>**ストラテラ ®**
効果	不注意や多動・衝動の症状が軽快し生活や仕事がしやすくなる	
効果発現	飲んだ**その日**	始めて **3**週間たって
効果持続	約 **12** 時間	約 **24** 時間
形	錠剤	カプセル
飲み方	**朝 1** 回	1日 **1** or **2** 回 **朝**or/and **夜**
主な副作用	食欲の低下 **41** %<br>動悸 **23** %<br>体重の減少 **21** %<br>眠れない **19** %<br>吐き気 **18** %<br>口が渇く **16** %<br>頭痛 **12** %	吐き気 **48** %<br>食欲の低下 **22** %<br>眠気 **18** %<br>口が渇く **15** %<br>頭痛 **12** %
処方	登録医のみ処方できる<br>（処方できる医師や施設を紹介する）	—
費用<br>自己負担額は保険種別で異ります	18mg錠 338円, 27mg錠 375円<br>36mg錠 403円<br>（1日18mg～72mgで調節）<br>例. 自己負担3割の場合：<br>**1**日**100**円～**240**円程度	40mgｶﾌﾟｾﾙ 462円<br>（1日40～120mgで調節）<br>例. 自己負担3割の場合：<br>**1**日**140**円～**420**円程度

どちらの薬を選ぶ？

●考えを整理しましょう

あなたにとって、以下の薬の特徴は、どの位重要ですか？
0～5で重みづけをしてみましょう。

内容	重要でない					重要である
効果が現れるまでの時間	0	1	2	3	4	5
効果が持続する時間	0	1	2	3	4	5
形状	0	1	2	3	4	5
回数や時間など薬の飲み方	0	1	2	3	4	5
副作用	0	1	2	3	4	5
費用	0	1	2	3	4	5

その他（気になること）

•	0	1	2	3	4	5
•	0	1	2	3	4	5
•	0	1	2	3	4	5
•	0	1	2	3	4	5
•	0	1	2	3	4	5

● 話し合う準備をします

　あなたの重みづけや考えをもとに「薬を飲む or 飲まない」「飲む場合どちらを選ぶ」かについて、次の診察で話し合います。準備はできましたか？

疑問や気になったことを書いておきましょう

おわりに

●自分にあった対処法・治療法を選ぶために

治療の選択肢には、それぞれ長所と短所があります。この手引きは、それらをよく理解し、自分にとって重要なことを明らかにしながら医療者と話し合い、あなたに合った選択ができるよう作られています。

●手引きの開発プロセス

この手引きは、社会人になってADHDとわかった当事者の方々の声や意見をもとに作成しました。また、精神科の専門家のチェックも受けています。なお、企業などからの資金援助は受けていません。

●手引きの更新

この手引きは、必要に応じて見直しと更新をおこないます。

※ ここに掲載された情報は、医療者と話し合いながら対処法や治療法を決める際の手引きとなるものであり、医療者のアドバイスの代わりになるものではありません。

引用文献・参考資料

1) 米精神医学会. 精神疾患の診断・統計マニュアル DSM-5. 医学書院, 2014

2) 樋口輝彦，齊藤万比古 監修．成人期ADHD診療ガイドブック．じほう，2013

3) Kessler RC. Et al., Am j Psychiatry, 163(4):716-23, 2006

4) Simon V. et al., Br J Psychiatry, 194:204-11, 2009

5) 齊藤 卓弥. 臨床精神医学, 46(10) : 1233-42, 2017

6) メチルフェニデート塩酸塩徐放錠 添付文書 http://www.pmda.go.jp

7) アトモキセチン塩酸塩カプセル 添付文書 http://www.pmda.go.jp

8) 岡田俊. 精神医学, 59(3):253-258, 2017

9) 渡邉 衡一郎, 神経系に作用する薬剤 In 今日の治療薬 2019. 南江堂, 2019.

作成：青木裕見 聖路加国際大学看護学研究科

作成日：2019年1月20日　　更新予定日：2021年1月

平成29・30年度 文部科学研究費補助金（研究活動スタート支援・研究代表者 青木裕見）による助成により作成されたものです。

©2019, Yumi Aoki 無断複写・無断転載はご遠慮ください

For individuals who found out that they have attention deficit hyperactivity disorder after becoming an adult

A decision aid to finding the appropriate coping and treatment methods

About this Decision Aid

Attention deficit hyperactivity disorder (ADHD) is a condition that is characterized by inattention and hyperactivity. ADHD in children, typically observed as "children who move around in class," is commonly encountered, but even adults may have ADHD symptoms, whose various aspects of their lives have been affected by these symptoms. There are also adults who experience problems in the workplace due to their symptoms.

Firstly, we describe the characteristics of “adults with ADHD” and the strategies and measures that could be used to manage the symptoms. Further, we have summarized the drug treatments that can be used in addition to the coping methods. You should understand your symptoms/characteristics and use this decision aid when you and your healthcare professional are trying to choose the right treatment method for you.

How to use this Decision Aid

This decision aid is designed to help adults with ADHD in selecting the appropriate treatment while consulting with a healthcare professional. It should be brought home by the individual and read carefully. This decision aid will be useful for both individual and healthcare professional who are working together to carefully identify the best treatment strategy.

Read the decision aid carefully

Check those that apply and make
a comment in the memo column

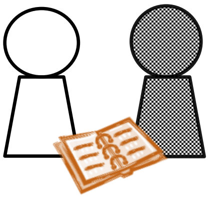

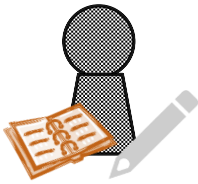

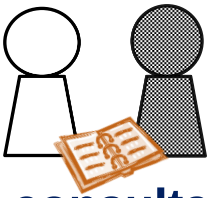

During consultation

You and your doctor share

- Your current condition
- The coping/treatment options

During consultation

- The checked items are noted and discussed
- The further treatment strategy is decided

If unable to decide at once, the decision aid should be brought home to help you in making a decision.

Table of Contents

- Get to Know ADHD 5
- Finding coping methods 11
- Drug treatments that can be used in addition to the coping methods 15

Get to Know ADHD

Do you ever experience this?

● When instructed by your boss

☐ The instruction is not retained in your head, if the instruction was given verbally only (it is better to have it written).

☐ You can't remember what was said first.

☐ When a lot is said all at once, your mind goes blank.

● When you answer the phone

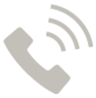

☐ You misunderstand the message or request.

☐ You forget to ask who is on the other line.

● While working in the office

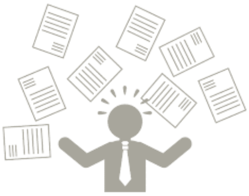

☐ You enter a name or number incorrectly.

☐ You skip reading what is written.

☐ You perform the task even though you have not understood the instructions, leading to failure in properly accomplishing the task.

☐ Your boss reminds you that ‘there is no progress report’.

● At meetings

☐ You are tapping your feet unconsciously, clicking your pen, or fidgeting.

☐ You doze off on a topic that you are not interested in.

Do you ever experience this?

● During conversations with colleagues

☐ You cannot remember the conversations if asked “How was that?”

☐ Although it will eventually be revealed, you tell a lie or say something inappropriate.

☐ You get agitated easily.

☐ You can be friendly, but feel tired later.

● When doing deskwork

☐ You soon want to go to a different place and take a break.

☐ You find it stressful to sit down, except when the activity is something that you are interested in.

● When performing a task that has been set up

☐ You are reminded to not act at your own discretion.

● When you get promoted

☐ You cannot manage your subordinates well.

The experiences described here are of those individuals who found out that they have ADHD after becoming members of the society. Do you have any similar experience?

☞ Next, we will look at the characteristics of ADHD.

Characteristics of ADHD in Adults

Carelessness and hyperactivity/impulsivity are two symptoms[1)]

Carelessness

- Careless mistakes are made easily
- It is difficult to maintain attention
- You find it difficult to organize tasks, and it is impossible to complete the tasks until the end.
- You easily forget what you need and even your daily activities
- You tend to be easily distracted by external stimuli

And so on

Hyperactivity/impulsivity

- Your hands and feet are restless and you are always fidgeting
- You cannot sit still in situations where you are expected to do so
- You cannot keep still
- You talk too much and start answering inadvertently before the question is over
- You are not good at waiting for your turn
- You are likely to disturb or interrupt other people

And so on

Characteristics of ADHD in Adults

What is the cause?

ADHD is caused by an unbalanced (uneven) functioning of the brain and the neurotransmitters in the brain are affected[2].

Am I the only one with this condition?

No, ADHD is absolutely not an unusual condition. In fact, 2-4 of every 100 adults are said to have ADHD symptoms[3][4].

Am I only presenting ADHD symptoms?[5]

Approximately 70% of individuals with adult ADHD have other psychiatric disorders. In particular, an increasing number of individuals may experience mental health problems because of work challenges and are diagnosed with depression. There are also many individuals with ADHD who have autism spectrum disorder, which are classified as the same developmental disorder, or bipolar disorder, which are characterized by repeated episodes of mania and depression.

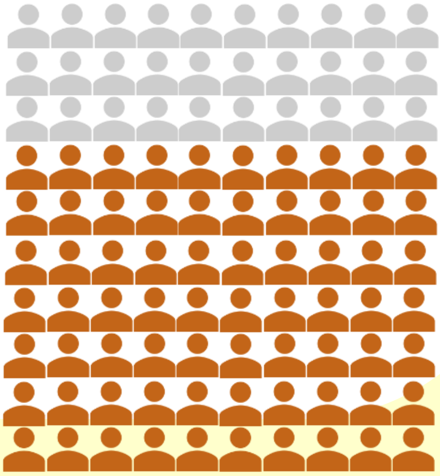

About 30 out of 100 people with adult ADHD have no other psychiatric disorders (Absent).

About 70 out of 100 people with adult ADHD have other psychiatric disorders (Present).

Characteristics of ADHD in Adults

● Is this a disability?

Although ADHD is classified as one of the developmental disorders, it is not permanent even if it is called a disorder. The symptoms of ADHD can cause problems, especially when an individual experiences difficulties in work and life. Thus, by knowing your own characteristics and devising solutions, the symptoms may become less noticeable.

Write your doubts and questions about ADHD

☞ Next, we will look at the coping methods.

Finding the best coping methods

Finding coping methods

Once you have understood the characteristics of ADHD, the next thing to do is

1. Know your characteristics

The first step is to get to know yourself. What are the things you find dificcult to do? When did you notice it? On the other hand, what do you like? What are you good at? What do you find enjoyable doing?

You can ask the people around you. Please write them down.

[Things you find difficult to do]

[Things you like and enjoy doing]

2. Making countermeasures

Next, you will make countermeasures

・You easily forget things	⇒	Keep the things you need in one place
・You frequently have miswords when talking	⇒	Count 10 seconds before speaking
・You cannot meet deadlines	⇒	Use reminders on your smartphone
・You cannot keep things	⇒	Decide where to put them. Throw away things that you don’t use.
・You came up with a good idea	⇒	Think about it overnight
・You cannot organize your schedules	⇒	Set aside 30 minutes of free time in between tasks
・Eliminate written notes	⇒	Use only one notebook and carry it around with you
・You don’t know how to prioritize	⇒	Use color-coded or numbered information

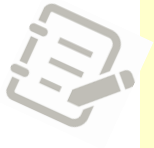

It is also important to ask help from other people...

・You make many careless mistakes	⇒	Ask someone to double check it for you
・You cannot meet deadlines	⇒	Ask someone to remind you verbally
・You can't remember the instructions	⇒	Write them down and ask someone if they are correct

What else can you do?
Let’s look back on your characteristics and make countermeasures that you will put into practice.

[Making countermeasures]

Let us come up with a coping method that you might be able to do

Example: Use to-do-list stickers and place them in areas where you can regularly see them.

Drug treatments that can be added to the coping methods

In addition to the coping methods, medications can also be taken

In adults with ADHD, apart from learning and using coping methods that you have devised and asking help from people around you, there is also the option of taking medications. Here, we will identify the advantages and disadvantages of taking and not taking medications.

	Take medications	Not take medications
Advantage	● Symptoms improve [6-9]<br>You become calm and more careful<br>☞ It becomes easy to perform daily life and work activities	● Side effects can be avoided<br>● No burden of cost
Disadvantages	● 76-80 out of 100 people experience some side effects [6)7)]<br>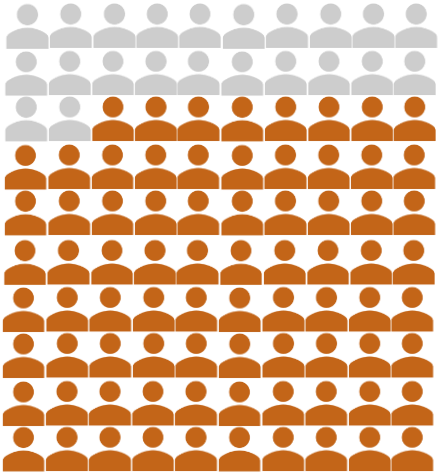<br>Details of the side effects, such as nausea, decreased appetite, and sleep disorders are on page 18<br>● Costly<br>(the amount paid varies depending on the individual's type of insurance)	● If the symptoms do not improve by the coping methods alone, you will continue to experience them<br>☞ The symptoms will interfere with the performance of daily life and work activities

In addition to the coping methods, medications can also be taken

Organize your thoughts

How important is the following items to you in terms of taking medications? Score each item using a 0-5 scale with 0 indicating not important and 5 indicating important.

Contents	Not important					Important
ADHD symptoms are improved	0	1	2	3	4	5
Daily life and work activities have become easier to perform	0	1	2	3	4	5
Side effects can be avoided	0	1	2	3	4	5
There is no burden of cost	0	1	2	3	4	5
Other things of concern						
•	0	1	2	3	4	5
•	0	1	2	3	4	5
•	0	1	2	3	4	5
•	0	1	2	3	4	5

☞ Next, we will look at the medication options if you want to receive drug treatments.

– For those who want to take medications -

Which drug to use?

There are two drug options. Let us compare the features of both drugs [6-9].

	Methylphenidate Hydrochloride Slow-Release Tablets<br>Concerta ®	Atmoxetine Hydrochloride<br>Strattera ®
Effect	Symptoms of carelessness, hyperactivity, and impulsivity are improved, and performing daily life and work activities become easier	
Onset of the effect	On the day that you took the drug	At 3 weeks after taking the drug
How long does the effect lasts?	Approximately 12 hours	Approximately 24 hours
Form	Tablet	Capsule
How to take it	Once in the morning	Once or twice daily (in the morning and/or evening)
Main side effects	Nausea **48** %<br>Decreased appetite **22** %<br>Drowsiness **18** %<br>Thirst **15** %<br>Headache **12** %	Decreased appetite **41** %<br>Palpitations **23** %<br>Weight loss **21** %<br>Sleeping problems **19** %<br>Nausea **18** %<br>Thirst **16** %<br>Headache **12** %
Prescribing	Only registered physicians can prescribe it (we will introduce the doctors and institutions that can prescribe it)	—
Cost (The copayment varies according to the insurance type)	18-mg tablet, ￥338;<br>27-mg tablet, ￥375;<br>36-mg tablet, ￥403.<br>(the dose is adjusted to 18-72 mg daily) Example: In the case of a 30% copayment, about ￥100-240 per day is spent	40-mg capsule, ￥462<br>(the dose is adjusted to 40-120 mg daily) Example: In the case of a 30% copayment, about ￥140-420 per day is spent

Which drug to choose?

Organize your thoughts

How important is the following items to you in terms of taking medications? Score each item using a 0-5 scale with 0 indicating not important and 5 indicating important.

Content	Not important					Important
Time for the effect to appear	0	1	2	3	4	5
How long the effects lasts	0	1	2	3	4	5
Form	0	1	2	3	4	5
Frequency and timing of taking the medication	0	1	2	3	4	5
Side effects	0	1	2	3	4	5
Cost	0	1	2	3	4	5
Other things of concern						
•	0	1	2	3	4	5
•	0	1	2	3	4	5
•	0	1	2	3	4	5
•	0	1	2	3	4	5
•	0	1	2	3	4	5

Get ready to discuss

Based on your understanding, in the next consultation, we will discuss whether you will take or not take medications.

If you decide to take medications, which one will you choose?

Are you ready for discussion?

Write down your questions and concerns

Conclusion

● To determine the right coping and treatment methods that are best for you

Each treatment option has its advantages and disadvantages. This decision aid is designed to help you understand these options well, discuss them with your healthcare provider while determining what is important to you, and make the choices that are right for you.

● Development of the decision aid

This decision aid was developed based on the responses and opinions of people who were diagnosed with ADHD after becoming an adult. The decision aid has also been checked by psychiatric specialists. In addition, we have not received financial support from companies.

● Updating the decision aid

This decision aid will be reviewed and updated as necessary.

The information provided here is intended to guide you in identifying the right coping or treatment method for you while you are consulting with a healthcare professional; however, this decision aid is not intended to replace the advice provided by the healthcare professional.

References

1) American Psychiatric Association. Diagnostic and Statistical Manual of Mental Disorders: DSM-5. Amer Psychiatric Pub Inc; 2013

2) Teruhiko Higuchi and Kazuhiko Saito. Supervising Editors. Adult ADHD Medical Guidebook. Jiho, 2013

3) Kessler RC, et al. Am J Psychiatry, 163(4):716-23, 2006

4) Simon V, et al. Br J Psychiatry, 194:204-11, 2009

5) Takuya S. Clinical Psychiatry, 46(10):1233-42, 2017

6) Methylphenidate hydrochloride tablets package insert. Available from http://www.pmda.go.jp

7) Atmoxetine hydrochloride capsule package insert. Available from http://www.pmda.go.jp

8) Takashi Okada, Psychiatry, 59(3):253-258,2017

9) Koichiro Watanabe. Medication on the nervous system in today's therapeutics 2020. Nankodo, 2020

Written by: Yumi Aoki, Graduate School of Nursing,

St. Luke International University

Date created: January 20, 2019

Scheduled to be updated on: January, 2021

This decision aid was developed with a grant from the 2017/2018 Ministry of Education Research Grant Program (Grant-in-Aid for Research Activity Start-up; the principal investigator is Yumi Aoki).

©2019, Yumi Aoki
